# Supplementary material for: Uncharacterized protein C17orf80 – a novel interactor of human mitochondrial nucleoids
Source: J Cell Sci. 2023 Jul 31;136(15):jcs260822. doi: 10.1242/jcs.260822 (PMC10445727; doi:10.1242/jcs.260822)
Supplement: Supplementary information [file joces-136-260822-s1.pdf]

A

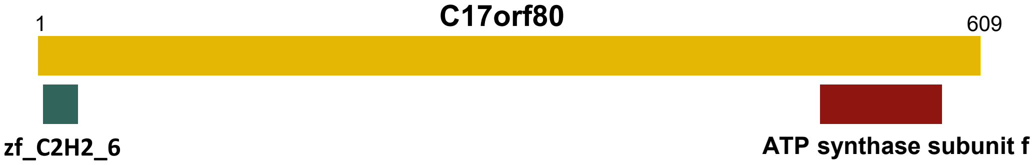


```
6J5I_f ATP synthase subunit f, mitochondrial; membrane protein; HET: ADP, ATP; 3.34A {Sus scrofa}
Probability: 99.54%,   E-value: 2.6e-15,   Score: 128.93,   Aligned cols: 82,   Identities: 26%,   Similarity: 0.437,   Template Neff: 9.3
Q ss_pred                ccCCcccccccccccCCCCchhhcCCCCchHHHHHHHHHheeE---EEeeecCCCccHHHHHHhheeEeeeeecchHHHH-hhcCCC
Q ENSP0000044055   503   SQVSLLERSSTHIRSLEPPAGLTTSNFSLMRLLGAVQKGWIR---CNTTIRKSGFGGITMLFTGYFVLCCSWSFRRLKK-LCRPLP   584 (609)
Q Consensus        503   ~qgpL~eRsl~dVRl~ElP~WLat~~~S~pRll~avqk~W~w---YYidVKKGGVGGItMLlAGYCVLsY~WSY~HlK~-~WRkyh   584 (609)
                         +++|+.++.++||+++|+|.||..++++..+...++.+.|.+ -||++++++++||.|+++|+|||+|+|+|+|++. +|+|||
T Consensus          2     ~~~~~~~~~~~~~k~~e~p~w~~~r~~~~~~~~~~~~~~r~~~~~y~~ky~~~~~~~~~~~~~~~~~~~~~~~~y~~~~y~~~~~~~~~~kyh   87 (87)
T 6J5I_f             2     SVVPLKDRRLLEVKLGELPSWILMRDFTPSGIAGAFQRGYYRYYNKYVNVKKGSVAGLSMVLAAYVVFNYCRSYKELKHERLRKYH   87 (87)
T ss_dssp                CHHHHHHHHHHHHHHHTCC-------CCTTHHHHCSHHHHGGGSCSCCCCTTTSCHHHHTTHHHHHHHHHHHHHHTTTCCCCCSSCCC
T ss_pred                CCCCCCCCCcccCccccchhHHHhccCCCHHHHHHHHHHhchhhhhhcCcCCCChHHHHHHHHHHHHHHHHHHhcHHHHHHHhccC
```

```
PF18450.4 zf_C2H2_6 ; Zinc Finger domain
Probability: 91.33%,   E-value: 0.097,   Score: 37.16,   Aligned cols: 21,   Identities: 33%,   Similarity: 0.729,   Template Neff: 4.1
Q ss_pred                    ccCCCCCCchhh---cccCCCccc
Q ENSP0000044055       9    EVCPYCKKPFKR---LKSHLPYCK   29 (609)
Q Consensus            9    EvCPyCgKpFKR---LKSHLPHCK   29 (609)
                              .+||||++.|-+ ++-|.-||.
T Consensus            3    ~~CpyC~lrF~sp~~k~~He~~C~   26 (28)
T PF18450.4            3    TVCIYCSLRFSSLELKHDHEITCE   26 (28)
T ss_pred                    ccccccCCccccChHHHHHHhhcc
```

B

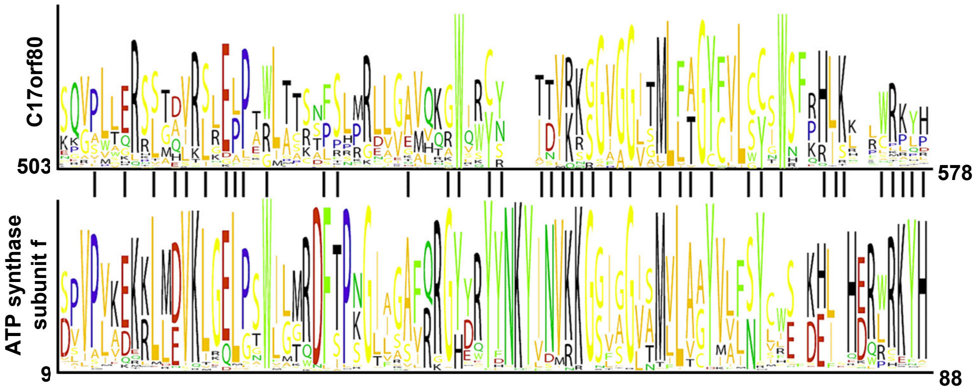

Fig. S1. C17orf80 homology analysis. A. HHPred search results. Schematic representation of detected homology domains followed by alignments of C17orf80 with ATP synthase subunit f and a zinc finger domain. **B**. Sequence logos of C17orf80 C-terminus and vertebrate ATP synthase subunits f showing conservation of the region. Vertical lines between the template and query indicate similar and identical residues.

Journal of Cell Science • Supplementary information

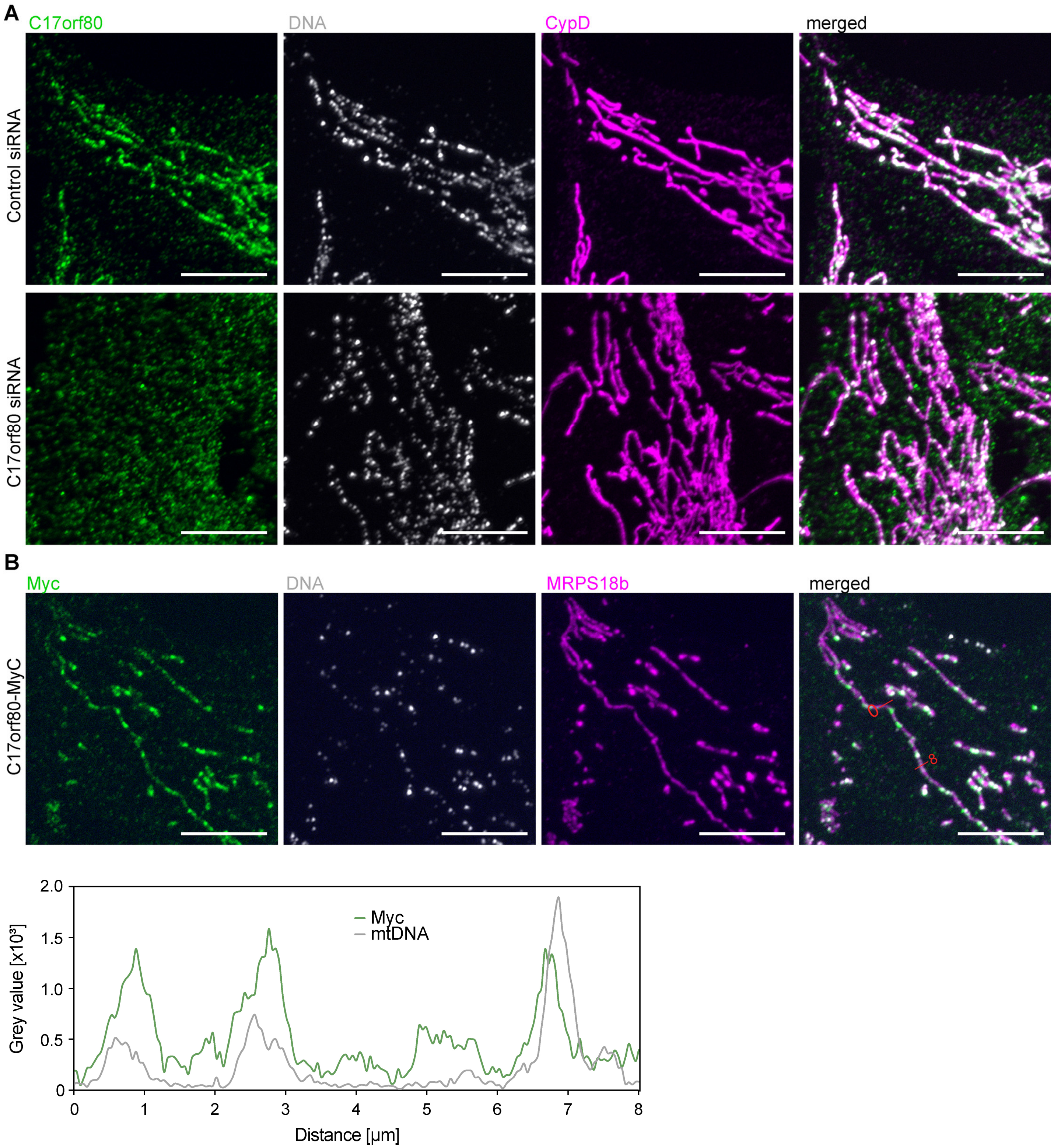

Fig. S2. C17orf80 antibody validation. A. siRNA-mediated silencing of C17orf80. U2OS cells were treated with negative control or C17orf80-specific siRNAs. C17orf80 immunofluorescence was observed in the mitochondrial network and cytoplasm, however, the cytoplasmic signal does not relate to C17orf80. Co-immunostaining with C17orf80, CypD (mitochondrial network), and DNA (nucleoids and nuclei) antibodies. The scale bar is 10 μm. **B**. Transiently expressed cMyc-tagged C17orf80 shows both uniform signal and foci that colocalize with mtDNA. U2OS cells were transfected with pCMV6-Entry-C17orf80-cMyc plasmid for 48 hours. Co-immunostaining with Myc-tag, MRPS18b (mitochondrial network), and DNA (nucleoids and nuclei) antibodies. The scale bar is 10 μm. The plot represents fluorescence intensities profiles of the individual channels along a 8 μm line; the ends of the line are marked on the merged image.

Journal of Cell Science • Supplementary information

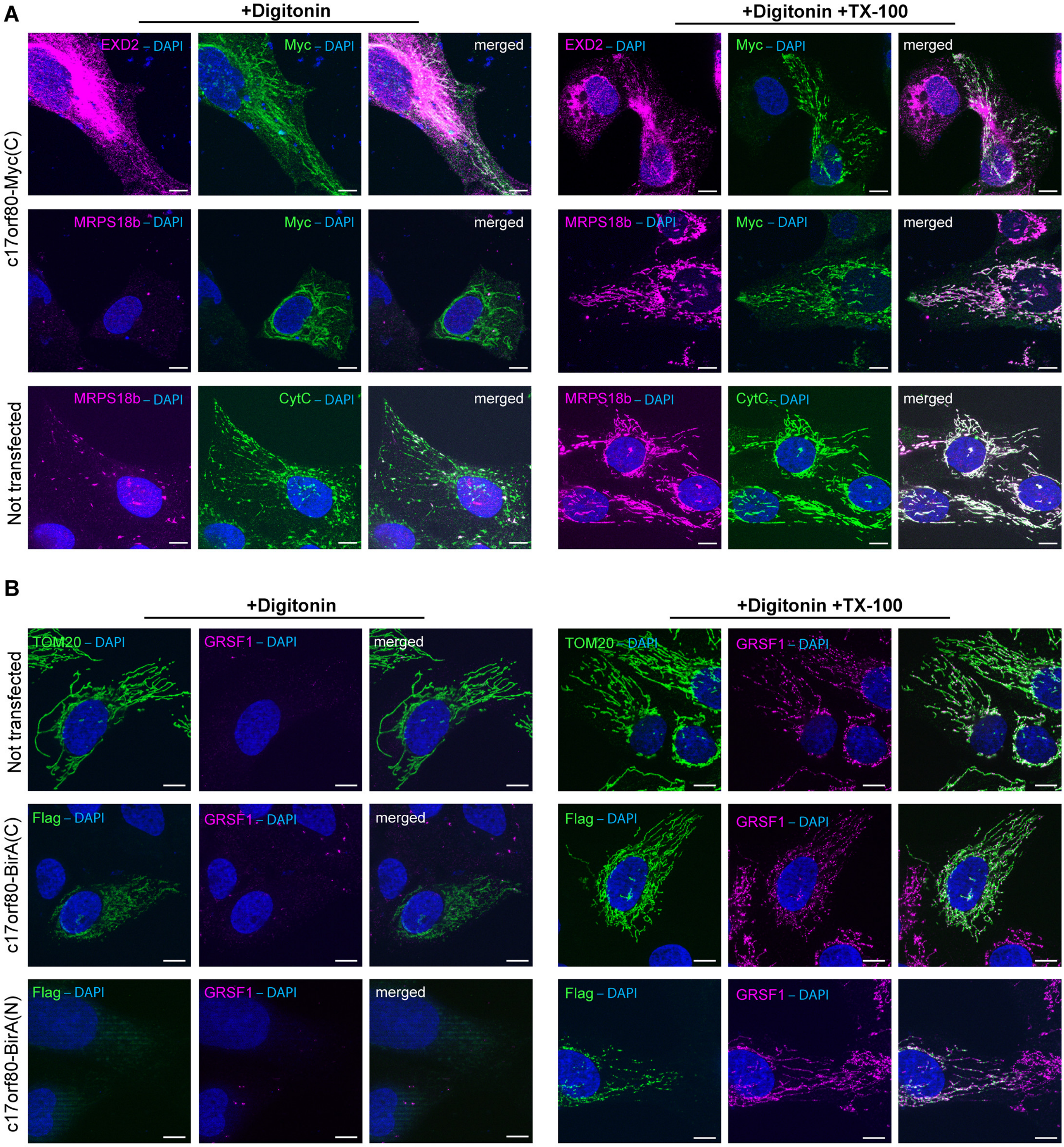

Fig. S3. Antibody accessibility assay performed with cells overexpressing tagged C17orf80. A. The C-terminal Myc-tag is partially accessible for the antibody binding without permeabilization of IMM. U2OS cells were transfected with pCMV6-Entry-C17orf80-cMyc plasmid, permeabilized with either digitonin or a combination of digitonin and TX-100 and co-labelled with antibodies against Myc-tag and EXD2 (OMM) or MRPS18b (matrix). Co-labelling with EXD2 was used to confirm that the observed signal was mitochondrial, as we could not use the combination of Myc-tag and TOM20 antibodies; the specificity of the EXD2 antibody has been validated previously *(Hensen et al., 2018)*. The IMS protein Cytc was used to test for IMS accessibility. Nuclei stained with DAPI. The scale bar is 10 μm. **B.** The C-terminal Flag-tag of the C17orf80-BirA*(C) fusion is partially accessible for the antibody binding without permeabilization of IMM. We did not detect any signal derived from the N-terminal Flag-tag of the C17orf80-BirA*(N) fusion in cells permeabilized with digitonin only. U2OS cells were transfected with pDEST-pcDNA5-C17orf80-BirA*-FLAG N- or C-term plasmids, permeabilized with either digitonin or a combination of digitonin and TX-100 and co-labelled with antibodies against Flag-tag or TOM20 (OMM), and GRSF1 (matrix). Nuclei stained with DAPI. The scale bar is 10 μm.

Journal of Cell Science • Supplementary information

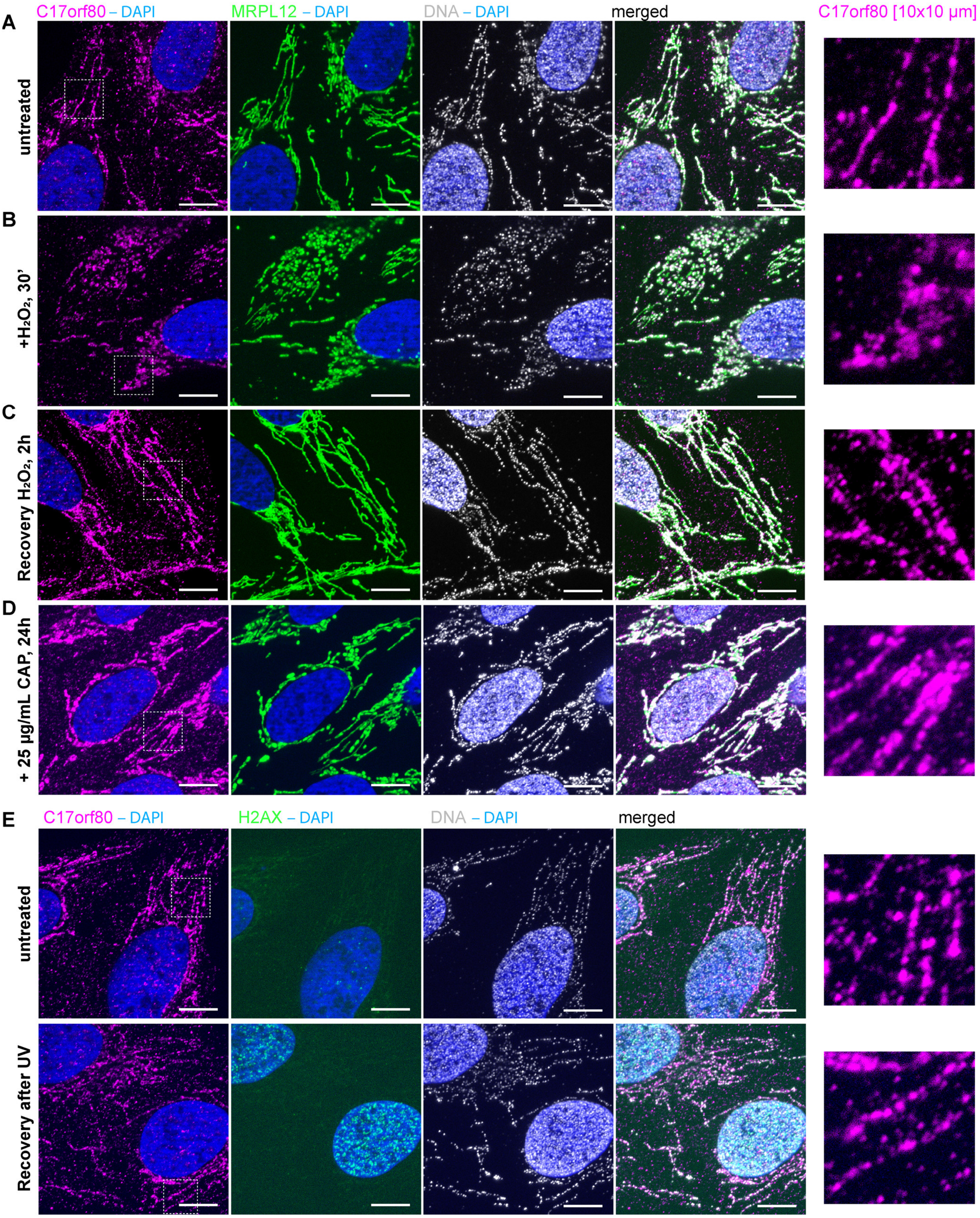

Journal of Cell Science • Supplementary information

Fig. S4. Sub-mitochondrial localization of C17orf80 in cells undergoing different types of stress. A. Untreated U2OS cells. **B.** Cells were treated with 200 µM H2O2 for 30 min displayed a fragmented mitochondrial network and more diffused C17orf80 signal. **C.** After 2 h of recovery in fresh medium, the cells displayed hyperfused mitochondrial network; the C17orf80 signal was back to its normal pattern. **D.** Cells were treated with 25 µg/mL chloramphenicol for 24 h displayed altered mitochondrial morphology and more diffused C17orf80 signal. **A-D.** Co-immunostaining with C17orf80, MRPL12 (mitochondrial network), and DNA (nucleoids and nuclei) antibodies. **E.** C17orf80 signal was not affected in cells recovering after exposure to ultraviolet light (UV) irradiation. Cells were exposed to 1.34 mJ/cm^2, 305-nm wavelength UVB for 30 sec and allowed to recover for 3 h. The immunofluorescence of H2AX histone was used as a control of the treatment efficiency as its signal increases in response to nuclear DNA damage *(Oh et al., 2011, Hensen et al., 2018)*. Co-immunostaining with C17orf80, H2AX, and DNA (nucleoids and nuclei) antibodies. **A-E.** Nuclei stained with DAPI. The scale bar is 10 *µ*m. Zoomed-in sections are 10x10 *µ*m.

Journal of Cell Science • Supplementary information

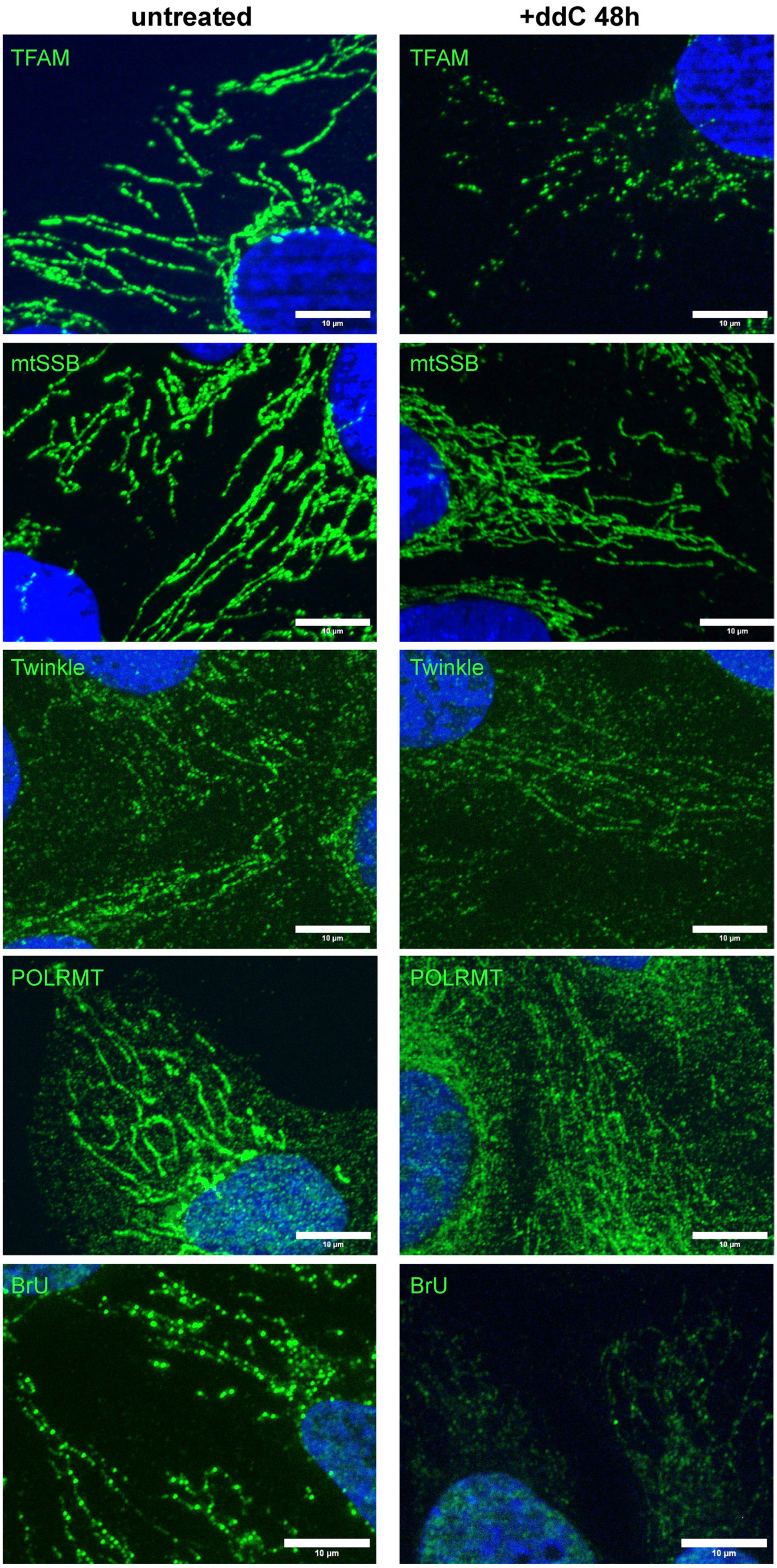

Fig. S5. Nucleoid-associated proteins and RNA granules before and after ddC treatment. IF images of ddC-treated and control cells stained with antibodies against TFAM, mtSSB, Twinkle or POLRMT, or anti-BrU (after 1 h BrU-labelling). Nuclei stained with DAPI (blue). The scale bar is 10 μm.

Journal of Cell Science • Supplementary information

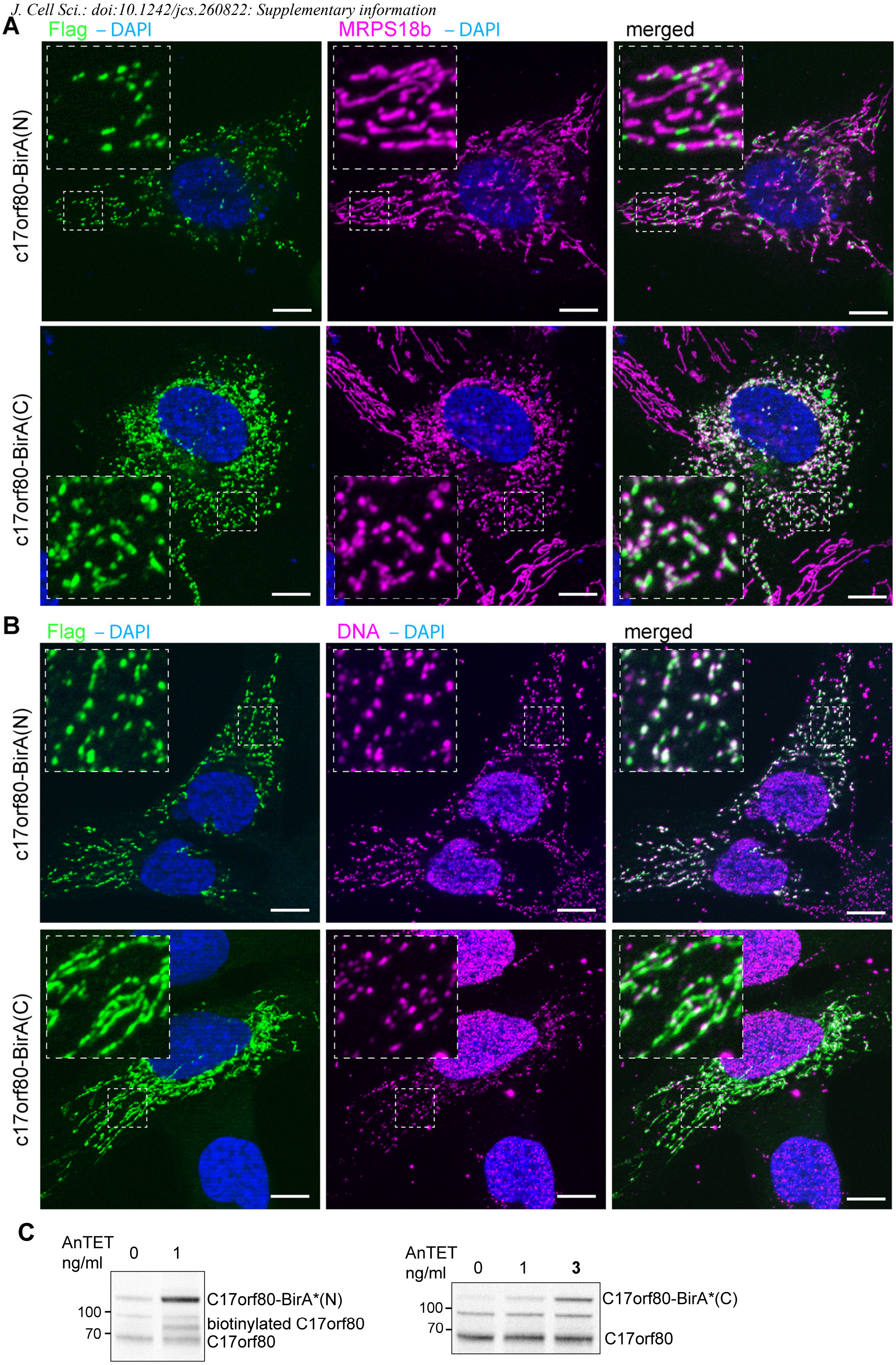
A
Flag – DAPI
MRPS18b – DAPI
merged
c17orf80-BirA(N)
c17orf80-BirA(C)
B
Flag – DAPI
DNA – DAPI
merged
c17orf80-BirA(N)
c17orf80-BirA(C)
C
AnTET ng/ml
0
1
100
70
C17orf80-BirA*(N)
biotinylated C17orf80
C17orf80
AnTET ng/ml
0
1
3
100
70
C17orf80-BirA*(C)
C17orf80

Fig. S6. Sub-mitochondrial localization of transiently expressed C17orf80-BirA* fusion proteins. U2OS cells were transfected with pDEST-pcDNA5-C17orf80-BirA*-FLAG N- or C-term plasmids. Co-immunostaining with Flag-tag and MRPS18b (mitochondrial network) or DNA (nucleoids and nuclei). **A.** Cells overexpressing C17orf80-BirA*(N) displayed normal morphology of the mitochondrial network; cells overexpressing C17orf80-BirA*(C) often had a fragmented mitochondrial network. **B.** The signal of C17orf80-BirA*(N) fusion was punctate and overlapped with mtDNA, while the signal of C17orf80-BirA*(C) fusion was more uniformly distributed throughout mitochondrial network. **A-B.** Nuclei stained with DAPI. The scale bar is 10 μm. Zoomed-in sections are 10x10 μm. **C.** Levels of C17orf80-BirA* fusion proteins after induction with AnTET as detected with the anti-C17orf80 antibody.

Journal of Cell Science • Supplementary information

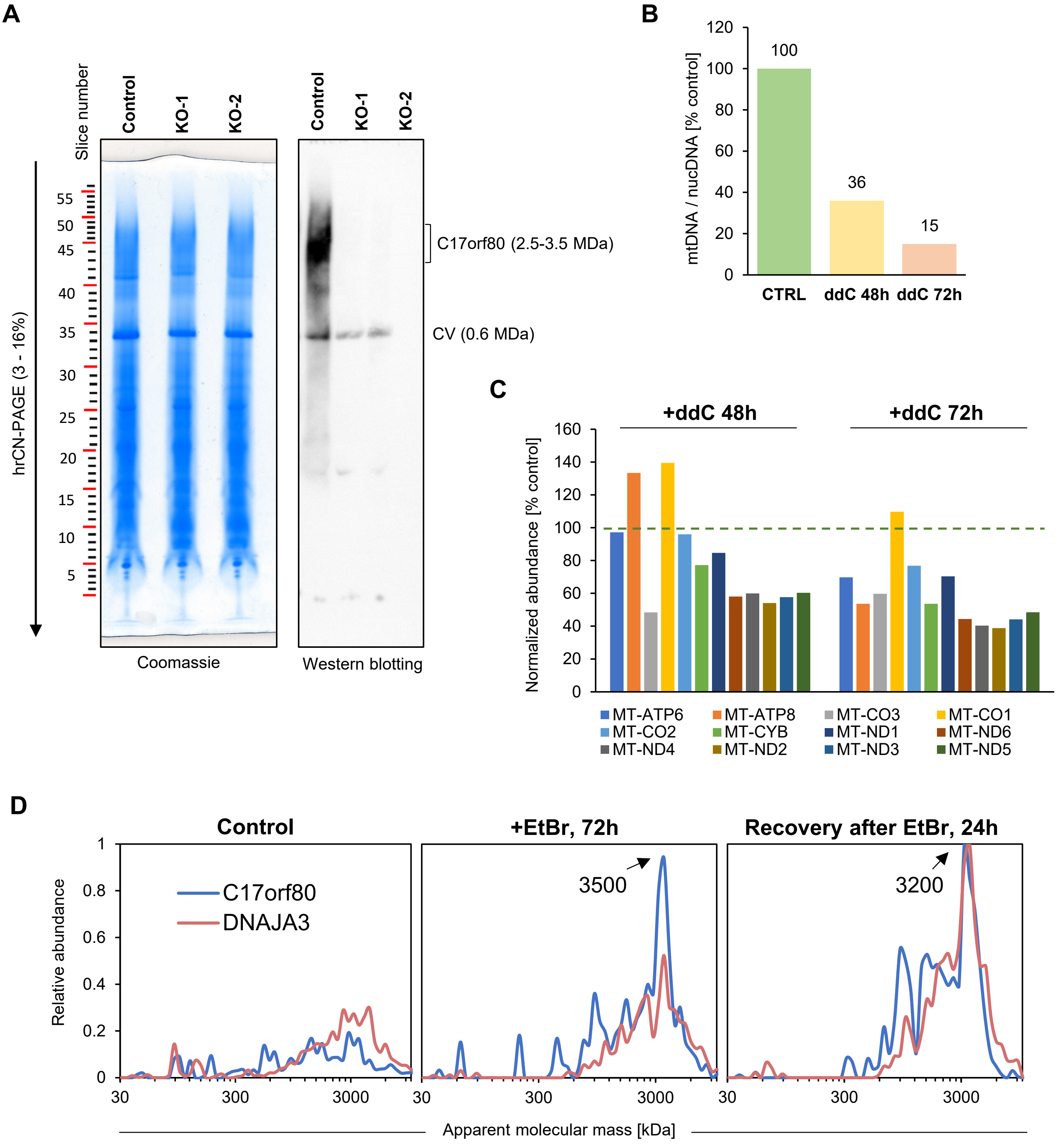

Fig. S7. Analysis of samples used for complexome profiling. A. C17orf80 migrates at a high molecular mass range when separated by native gel electrophoresis. Coomassie staining and Western blotting of parental control HEK293 cells and C17orf80 knockouts samples separated by the hrCNE are shown. The solubilized with digitonin mitochondrial samples (200 μg of protein) were loaded in two identical sets on a 3-16% native gel. After the run, one part of the gel was stained with Coomassie, while the other part was used for western blotting. The western blot was detected with the C17orf80 antibody. Note that Complex V non-specifically traps C17orf80 antibody. The scale on the right indicates the slices into which the gel was cut for MS analysis. **B.** The effects of ddC treatment. The relative mtDNA copy number decreased to 36% and 15% of that in control after 48 h and 72 h of treatment with 100 μM ddC as measured by qPCR (n = 1). **C**. Levels of the mtDNA-encoded OXPHOS subunits after ddC-treatment detected in the CP dataset. Total iBAQ intensities of ddC-treated samples are shown in relation to that of the control. MT-ND4L was not identified by MS in this dataset. **D.** Migration profiles of C17orf80 and DNAJA3 in control, EtBr-treated and recovering from EtBr samples retrieved from a published complexome dataset *(Potter et al., 2023).*

Journal of Cell Science • Supplementary information

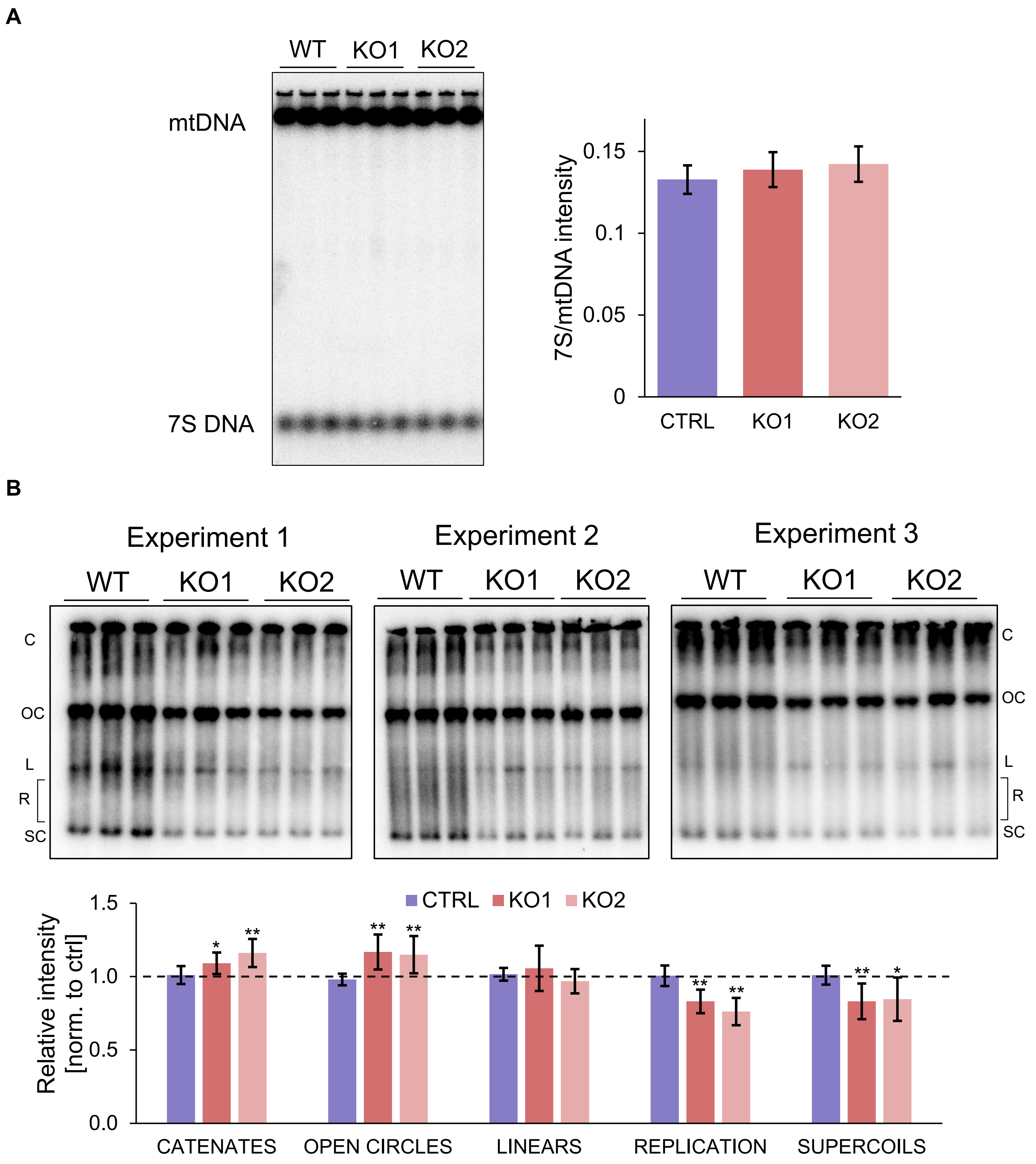

Fig. S8. C17orf80 knockouts. A. 7S DNA Southern blot and its quantification. No significant changes in 7S DNA levels were observed. Data are mean ± SD of the ratio between 7S DNA and mtDNA intensities of three biological replicates; two-tailed unpaired Student's t-test. **B.** Analysis of topological forms of mtDNA. KOs showed a 10-15% increase in catenates and open circles and a 15-25% decrease in supercoiled and replicating mtDNA molecules. Three independent Southern blots each containing three biological replicates of each cell line were analysed. The intensity of each topological form was measured in relation to the total intensity of the line. The first replicate of control was set to 1 for every topological form in each experiment. Data are mean ± SD of fold changes between control and KO from nine biological replicates; two-tailed unpaired Student's t-test. Cutoffs for statistical significance: **p* ≤ 0.05, ***p* ≤ 0.01. C = catenates (oligomeric mtDNA), OC = open circles, L = linear, R = replication, SC = supercoils.

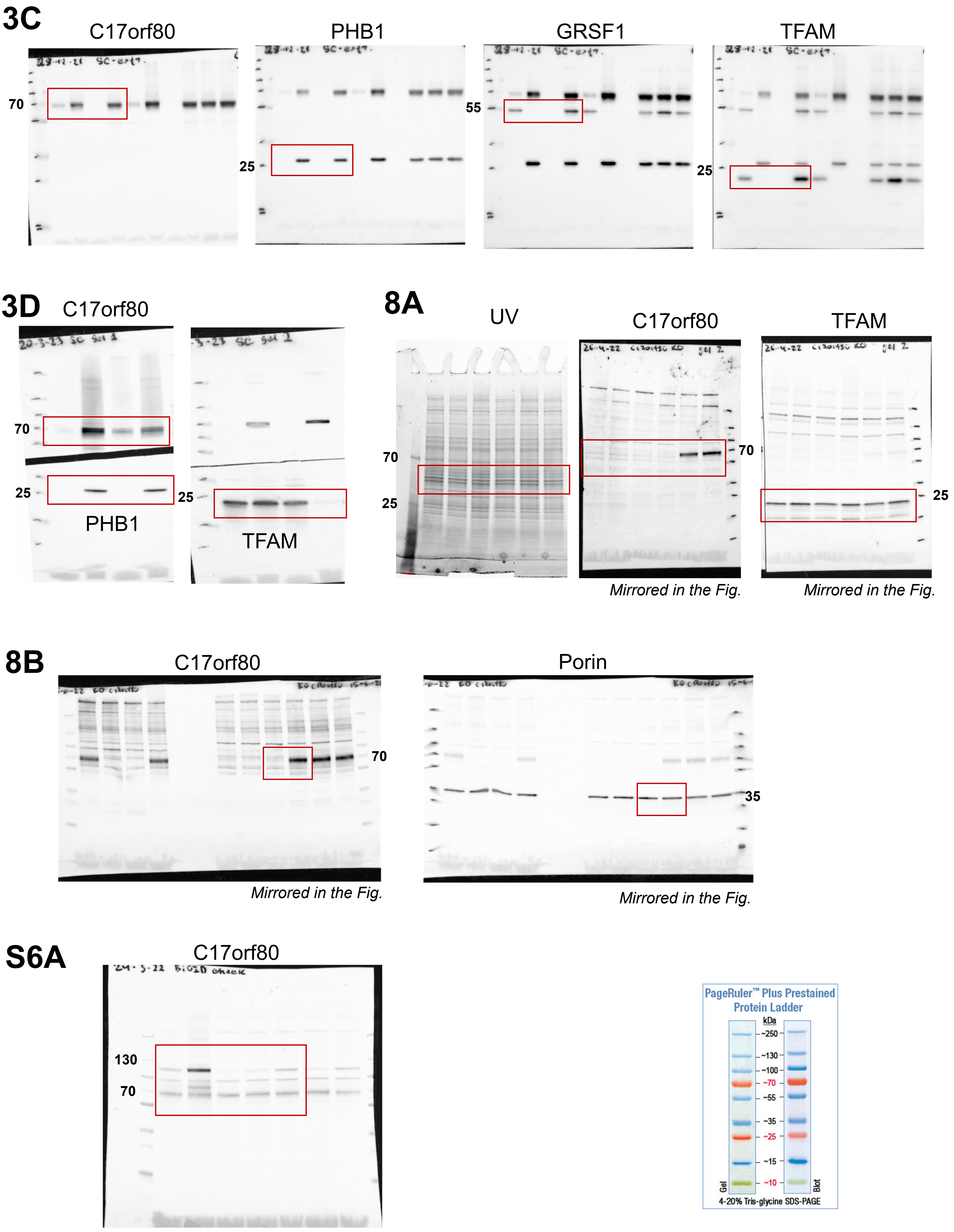

Fig. S9. Uncropped western blots.

Journal of Cell Science • Supplementary information

Table S1. Antibodies used for immunofluorescence (IF) and western blot (WB) detections

Antigen	Source / type	Manufacturer	Identifier	Dilution
**C17orf80**	Rabbit IgG	Proteintech	Cat #: 27762-1-AP<br>RRID: AB_2880964	WB 1:5000, IF 1:200
**TFAM**	Rabbit IgG	Gift of Dr. Rudi Wiesner	REF: *Pohjoismaki et al., 2006*	WB 1:10000
**Prohibitin 1**	Mouse IgG	Abcam	Cat #: Ab1836<br>RRID: AB_2164473	WB 1:5000
**GRSF1**	Rabbit IgG	Sigma-Aldrich	Cat #: HPA036985<br>RRID: AB_10672785	WB 1:10000, IF 1:200
**TFAM**	Mouse IgG	Abcam	Cat #: ab89818<br>RRID: AB_2043026	IF 1:200
**Cyclophilin F**	Mouse IgG	Abcam	Cat #: ab110324<br>RRID: AB_10864110	IF 1:400
**BrU**	Mouse IgG	Roche	Cat #: 11170376001<br>RRID: AB_514483	IF 1:50
**DNA**	Mouse IgM	Progen	Cat #: 61014<br>RRID: AB_2750935	IF 1:100
**TOMM20**	Mouse IgG	Santa Cruz	Cat #: sc17764<br>RRID: AB_628381	IF 1:100
**EXD2**	Rabbit IgG	Sigma-Aldrich	Cat #: HPA005848<br>RRID: AB_1078768	IF 1:200
**Cytochrome c**	Mouse IgG	BDPharmingen	Cat #: 556432<br>RRID: AB_396416	IF 1:100
**MRPL12**	Mouse IgG	Abcam	Cat #: ab58334<br>RRID: AB_944265	IF 1:400
**MRPS18b**	Rabbit IgG	Proteintech	Cat #: 16139-1-AP	IF 1:200
**mtSSB**	Rabbit IgG	Sigma	Cat #: HPA002866<br>RRID: AB_2146368	IF 1:200
**POLRMT**	Rabbit IgG	Abcam	Cat #: ab32988<br>RRID: AB_873619	IF 1:100
**Twinkle**	Mouse IgG	gift of Anu Suomalainen Wartiovaara	REF: *Rajala et al., 2014*	IF 1:50
**H2AX**	Mouse IgG	Merck Millipore	Cat #: 05-636-I<br>RRID: AB_2755003	IF 1:200

Journal of Cell Science • Supplementary information

Table S1. Antibodies used for immunofluorescence (IF) and western blot (WB) detections (continued)

Antigen	Source / type	Manufacturer	Identifier	Dilution
**Flag**	Mouse IgG	Sigma-Aldrich	Cat #: F1804<br>RRID: AB_262044	IF 1:400
**C-Myc**	Mouse IgG	Roche	Cat #: 11667203001<br>RRID: AB_390911	IF 1:1000
**Alexa Fluor 488 anti-rabbit IgG**		Invitrogen	Cat #: A-11008<br>RRID: AB_143165	IF 1:1000
**Alexa Fluor 488 anti-mouse IgG**		Invitrogen	Cat #: A-11001<br>RRID: AB_2534069	IF 1:1000
**Alexa Fluor 568 anti-mouse IgG**		Invitrogen	Cat #: A-11004<br>RRID: AB_2534072	IF 1:1000
**Alexa Fluor 568 anti-rabbit IgG**		Invitrogen	Cat #: A-11011<br>RRID: AB_143157	IF 1:1000
**Alexa Fluor 647 anti-mouse IgM**		Invitrogen	Cat #: A-21238<br>RRID: AB_2535807	IF 1:1000
**Alexa Fluor 647 anti-mouse IgG**		Invitrogen	Cat #: A-21235<br>RRID: AB_2535804	IF 1:1000

Table S2. C17of80 BioID

Click here to download Table S2

Table S3. Complexome profiling

Click here to download Table S3

Journal of Cell Science • Supplementary information

Supplemental references

Hensen, F., A. Moretton, S. van Esveld, G. Farge, and J.N. Spelbrink. 2018. The mitochondrial outer-membrane location of the EXD2 exonuclease contradicts its direct role in nuclear DNA repair. Sci Rep. 8:5368.

Oh, K.S., M. Bustin, S.J. Mazur, E. Appella, and K.H. Kraemer. 2011. UV-induced histone H2AX phosphorylation and DNA damage related proteins accumulate and persist in nucleotide excision repair-deficient XP-B cells. DNA Repair (Amst). 10:5-15.

Pohjoismaki, J.L., S. Wanrooij, A.K. Hyvarinen, S. Goffart, I.J. Holt, J.N. Spelbrink, and H.T. Jacobs. 2006. Alterations to the expression level of mitochondrial transcription factor A, TFAM, modify the mode of mitochondrial DNA replication in cultured human cells. Nucleic Acids Res. 34:5815-5828.

Rajala, N., J.M. Gerhold, P. Martinsson, A. Klymov, and J.N. Spelbrink. 2014. Replication factors transiently associate with mtDNA at the mitochondrial inner membrane to facilitate replication. Nucleic Acids Res. 42:952-967.
