# Supplementary material for: Uncharacterized protein C17orf80 – a novel interactor of human mitochondrial nucleoids
Source: J Cell Sci. 2023 Jul 31;136(15):jcs260822. doi: 10.1242/jcs.260822 (PMC10445727; doi:10.1242/jcs.260822)
Supplement: Supplementary file 1 [file joces-136-260822_review_history.pdf]

Uncharacterized protein c17orf80 - a novel interactor of human mitochondrial nucleoids

Alisa Potter, Anu Hangas, Steffi Goffart, Martijn A. Huynen, Alfredo Cabrera-Orefice and Johannes N. Spelbrink
DOI: 10.1242/jcs.260822

Editor: Jennifer Lippincott-Schwartz

Review timeline

Original submission:	23 November 2022
Editorial decision:	10 February 2023
First revision received:	30 April 2023
Accepted:	26 June 2023

Original submission

First decision letter

MS ID#: JOCES/2022/260822

MS TITLE: Uncharacterized protein c17orf80: a novel interactor of human mitochondrial nucleoids

AUTHORS: Alisa Potter, Anu Hangas, Steffi Goffart, Martijn A. Huynen, Alfredo Cabrera-Orefice, and Johannes N. Spelbrink
ARTICLE TYPE: Research Article

We have now reached a decision on the above manuscript.

To see the reviewers' reports and a copy of this decision letter, please go to: https://submit-jcs.biologists.org and click on the 'Manuscripts with Decisions' queue in the Author Area. (Corresponding author only has access to reviews.)

As you will see, the reviewers raise a number of substantial criticisms that prevent me from accepting the paper at this stage. They suggest, however, that a revised version might prove acceptable, if you can address their concerns. If you think that you can deal satisfactorily with the criticisms on revision, I would be pleased to see a revised manuscript. We would then return it to the reviewers.

Please ensure that you clearly highlight all changes made in the revised manuscript. Please avoid using 'Tracked changes' in Word files as these are lost in PDF conversion.

I should be grateful if you would also provide a point-by-point response detailing how you have dealt with the points raised by the reviewers in the 'Response to Reviewers' box. Please attend to all of the reviewers' comments. If you do not agree with any of their criticisms or suggestions please explain clearly why this is so.

© 2023. Published by The Company of Biologists under the terms of the Creative Commons Attribution License (https://creativecommons.org/licenses/by/4.0/).

Reviewer 1

Advance summary and potential significance to field

In this manuscript by Potter et al., the authors characterize a protein with a previously unknown functional role, c17orf80. This protein had previously been identified in proteomic datasets as a putative mitochondrial nucleoid interactor, though it had not been annotated as a mitochondrial protein in databases such as MitoCarta. The authors use immunofluorescence microscopy and biochemical assays to show that c17orf80 localizes to the interior of mitochondria and co-localizes with nucleoid components. They also show that mtDNA replication stress influences c17orf80 enrichment at nucleoid structures. The authors perform many additional experiments to ascertain a functional role for c17orf80, including complexome analysis, BioID, and also depleting c17orf80 using both CRISPR knockouts and siRNA and testing effects on mitochondrial mtDNA copy number and replication, for example. While extensive in number, these experiments ultimately failed to provide mechanistic insights into the role of this protein. In total, the manuscript successfully defines c17orf80 as a bona fide mitochondrial protein and convincingly demonstrates it co-localizes with nucleoids under stress conditions. The manuscript is clearly written and the authors acknowledge caveats of the experimental results.

Comments for the author

However, the biochemical experimentation further characterizing c17orf80 is preliminary as shown. The authors also present mass spectrometry-based data that hints at potential roles for c17orf80 without exploring these results in depth. The manuscript would be strengthened by improving topology data for c17orf80, including structure-function analysis, and further validation of complexome/proteomic results using complementary assays.

Specific points:

1. Experiments defining the submitochondrial localization/topology of c17orf80 are incomplete. The authors suggest that c17orf80 has two transmembrane domains and refer to Figure S1, though no data supporting this are shown. Two prediction programs (TMHMM and Phobius) both indicate c17orf80 has a single transmembrane domain, making it unclear if the zinc finger would face the matrix or intermembrane space. This also may explain the opposing results from N- and C-terminal BioID analysis. The carbonate extraction assays performed by the authors give inconclusive results, and the authors conclude on p.7 “This result agrees with the structural prediction of c17orf80 containing TM helices nevertheless indicating that it is not an integral membrane protein,” which is self-contradictory. The authors should more carefully determine the topology of c17orf80, perhaps with protease protection assays and also utilizing the tagged forms of the protein they have developed.
2. Basal enrichment of c17orf80 at nucleoid structures in untreated cells is not obvious in the images shown (fig. 2), and enlargements should be shown.
Linescan analysis would also be helpful to demonstrate that c17orf80 indeed enriches at nucleoid structures, both in these images and after ddC/EtBr treatment (Fig. 4).
3. The complexome analysis is not convincing given the low abundance of c17orf80 detected (19 peptides) and the high background (10 peptides identified in the KO!). To confirm that c17orf80 is in a high molecular weight complex, the authors should perform blue native PAGE or related analyses.
4. The potential interaction between DNAJA3 and c17orf80 is not explored in any depth, nor are hit proteins from BioID analysis. Blue native page analysis could be used to test if DNAJA3 co-migrates with c17orf80. Does loss of DNAJA3 affect c17orf80 migration size? The authors could also further confirm/explore a potential interaction between these and other proteins by immunoprecipitation and western.
5. If the putative zinc finger motif is indeed matrix localized, is this motif required for enrichment at nucleoid structures? A more careful structure/function analysis of the protein could be a way to enhance the manuscript.

© 2023. Published by The Company of Biologists under the terms of the Creative Commons Attribution License (https://creativecommons.org/licenses/by/4.0/).

Reviewer 2

Advance summary and potential significance to field

This manuscript describes the cytological, functional and biochemical characterization of a putative mitochondrial nucleoid protein, c17orf80. The work is generally well done and this is a useful contribution to the field.

Comments for the author

I encourage revision of this work to consider the following;
Essential to address:
1. Re evolutionary relationships of c17orf80, the text states that only potential homologs containing both zinc finger and ATP synthases homologous domains were considered. Are there other more distant homologs that contain one domain or the other?
2. Detection of the endogenous protein by immunofluorescence indicates that it is relatively abundant (comparable to e.g. TFAM). While it clearly colocalized with nucleoids and/or BrU foci under stressed conditions, the imaging as presented doesn't demonstrate a compelling homeostatic association.
3. I would appreciate a rationale for the fixation conditions use in Figure 3.
Could the authors provide some evidence that inner mitochondrial membrane structure is well-preserved?
4. The images presented in Fig 4 are too zoomed out. I can't easily see colocalization or lack thereof. The authors should add linescans showing fluorescence intensity for each channel, further zoomed in cropped images, or both.
5. In Fig 4 E there are horizontal lines across the cell nuclei which appear to be artifacts of either the microscopy or image processing. What is the explanation for this? Better quality images and raw data files need to be provided.
6. The methods re microscopy lack sufficient detail. What laser lines were used what is the pixel size, etc. See https://pubmed.ncbi.nlm.nih.gov/20513764/ Fig 2 as a starting point.
6. In the section 'C17orf80 accumulates in nucleoids upon short term treatment with ddC', data discussed including chloramphenicol, UV light and H_2O_2 treatment but are not shown. This is unacceptable. All data must be shown that are discussed.
7. Mitochondria appear fragmented upon overexpression of c17orf80-BirA-C term (Fig S5). Please discuss how this could contribute to the discrepancies in proteomic interaction networks between N term and C term tagged constructs presented in Fig 6.
8. The proper control for the proteomics presented in Fig 6 would be enrichment of nucleoid interactions as compared to a soluble matrix protein like eGFP. Was this normalization done? Perhaps I missed this.
9. The description of c17orf80 as a "nucleoid resident" protein in the Discussion is overstated, given the data.

Preferred, but not essential to address:
1. c17orf80 has two TM domains; I'm curious whether both are required for enrichment of the protein with nucleoid structures. Perhaps the authors could transiently express truncation mutant(s) from plasmids in the KO cells.

Reviewer 3

Advance summary and potential significance to field

In their work, Potter et al. define the localization of c17orf80 and endeavor to characterize its function. This protein has previously been classified as resident in mitochondrial RNA granules by high-throughput approaches coupling proximity labelling and mass-spectrometry. Using the same approach, the authors convincingly show that due to the predicted topology of c17orf80, N-terminal tagging of the protein (compared here with the previously employed C-terminal tagging) yields a more accurate assignment of c17orf80 as a peripheral component of mitochondrial nucleoids. This assignment is further supported by biochemical fractionation and mitochondrial complex profiling.

© 2023. Published by The Company of Biologists under the terms of the Creative Commons Attribution License (https://creativecommons.org/licenses/by/4.0/).

Immunofluorescence imaging using a commercial c17orf80 antibody shows the co-localization of the signal with mitochondria, TFAM and mitochondrial DNA. Interestingly, the authors observe that c17orf80 puncta increase in intensity when mtDNA replication is stalled by treatment with ddC, a cytosine analogue. Despite extensive investigation of the state of mtDNA following c17orf80 knock-out and knock-down, the authors havenÂ’t been able to assign a function for c17orf80 in cultured U2OS cells.

Comments for the author

Generally, I find this study well performed and data accurately interpreted. Given that it definitively addresses apparent inconsistencies in the high-throughput assignment of c17orf80, I recommend it for publication in JCS.

Major comments:
1. All immunofluorescence-based results in this study rely on the commercial c17orf80 antibody. Although the authors were able to validate the antibody using knock-down (Fig S2), they remark on variability in intensity and clear punctate background staining which persists after c17orf80 knock-down (Fig S2, p7). To strengthen key conclusions about c17orf80 localization, it is necessary to confirm the immunofluorescence data using an orthogonal imaging strategy. The authors could consider overexpressing a fluorescently tagged c17orf80, or perform immunofluorescence on the BirA-flag-fusion expressing cells.
2. The conclusion of this study could be strengthened by performing the following experiments, although these are not strictly required:
a. In addressing comment 1, the authors might consider performing the antibody accessibility test akin to Fig 3A on stained BirA-flag-fusion expressing cells. This could be a tractable way to experimentally test c17orf80s topology.
b. The authors remark on variability of c17orf80s behavior following carbonate extraction (p7), and later they observe c17orf80 relocalization following ddC treatment. It might be worthwhile to repeat the carbonate extraction following ddC treatment to assess whether replicative status might have contributed to the experimental variability shown in Fig 3.
c. The conclusion about c17orf80 interaction with DNAJA3 drawn from the mitochondrial complex profiling would be strengthened by direct IP. Although DNAJA3 but not c17orf80 was found to co-immunoprecipitate with nucleoids in the past, it is possible that their interaction could be detected biochemically.

Minor comments:
1. The authors should include data for intensity/relocalization of c17orf80 upon other mitochondrial stressors (rather than ‘data not shown’ on p9).
2. The authors quote the H(ES) value for c17orf80 but not DNAJA3 abundance variation in complex profiling performed in control vs ddC treated cells. This would be a helpful to support the conclusion that the two proteins interact.
3. The labels on top of Volcano plots in Fig 6A-B are confusing. Given the authors postulate there is a biologically significant interaction between c17orf80 and DNAJA3, it would be pertinent to label DNAJA3 in Figure Fig 6A-B in addition to clarifying the labels.
4. P13, line 20 refers to KO validation by western blot without a figure reference. Did the authors mean to refer to Fig 8A?
5. It would be helpful to include a color-code for Fig 7A in the figure as opposed to figure legend only. Additionally, c17orf80 should be differentiated from the pink protein-modifying enzymes.
6. Figure legends for Fig S8A and S8B are swapped.

First revision

Author response to reviewers' comments

We thank the reviewers for their feedback. We have included the suggested controls and data and,

© 2023. Published by The Company of Biologists under the terms of the Creative Commons Attribution License (https://creativecommons.org/licenses/by/4.0/).

subsequently, substantially re-crafted several parts of the manuscript and re-designed the figures. The corrected parts of the manuscripts are highlighted with blue in the PDF file.

Please find below the detailed replies to the reviewer's comments:

Reviewer 1 Advance Summary and Potential Significance to Field...

In this manuscript by Potter et al., the authors characterize a protein with a previously unknown functional role, c17orf80. This protein had previously been identified in proteomic datasets as a putative mitochondrial nucleoid interactor, though it had not been annotated as a mitochondrial protein in databases such as MitoCarta. The authors use immunofluorescence microscopy and biochemical assays to show that c17orf80 localizes to the interior of mitochondria and co-localizes with nucleoid components. They also show that mtDNA replication stress influences c17orf80 enrichment at nucleoid structures. The authors perform many additional experiments to ascertain a functional role for c17orf80, including complexome analysis, BioID, and also depleting c17orf80 using both CRISPR knockouts and siRNA and testing effects on mitochondrial mtDNA copy number and replication, for example. While extensive in number, these experiments ultimately failed to provide mechanistic insights into the role of this protein. In total, the manuscript successfully defines c17orf80 as a bona fide mitochondrial protein and convincingly demonstrates it co-localizes with nucleoids under stress conditions. The manuscript is clearly written and the authors acknowledge caveats of the experimental results.

Reviewer 1 Comments for the Author...

However, the biochemical experimentation further characterizing c17orf80 is preliminary as shown. The authors also present mass spectrometry-based data that hints at potential roles for c17orf80 without exploring these results in depth. The manuscript would be strengthened by improving topology data for c17orf80, including structure-function analysis, and further validation of complexome/proteomic results using complementary assays.

We agree with the reviewer that some of the presented data are preliminary. With this revision we have included more experimental validation of c17orf80 topology and added missing data and controls. We, however, considered that performing additional experiments in the direction of structure-function and validation of particular interactions is unreasonable considering the current state of our knowledge about this protein. We believe that this report is already valuable for the scientific community as it provides the first description of c17orf80 and gives the directions for the future research.

Specific points:

1. Experiments defining the submitochondrial localization/topology of c17orf80 are incomplete. The authors suggest that c17orf80 has two transmembrane domains and refer to Figure S1, though no data supporting this are shown. Two prediction programs (TMHMM and Phobius) both indicate c17orf80 has a single transmembrane domain, making it unclear if the zinc finger would face the matrix or intermembrane space. This also may explain the opposing results from N- and C-terminal BioID analysis. The carbonate extraction assays performed by the authors give inconclusive results, and the authors conclude on p.7 "This result agrees with the structural prediction of c17orf80 containing TM helices, nevertheless indicating that it is not an integral membrane protein," which is self-contradictory. The authors should more carefully determine the topology of c17orf80, perhaps with protease protection assays and also utilizing the tagged forms of the protein they have developed.

We thank the reviewer for pointing out the incorrect conclusion about the TM helices. We suggested the presence of two TM helices based on the homology with the ATP synthase subunit f, however, it was brought to our attention that according to the more recent ATP synthase structure, this subunit has only one TM-helix, while its second alpha-helix does not span the membrane. The TM-domain predictors indeed predict one TM helix in c17orf80. We have adjusted this information in the revised manuscript.

In this regard, to address the topology of c17orf80, we have performed an antibody accessibility

© 2023. Published by The Company of Biologists under the terms of the Creative Commons Attribution License (https://creativecommons.org/licenses/by/4.0/).

test using cells expressing either N- or C-tagged c17orf80 (i.e. Myc(C), BirA-Flag(C), BirA-Flag(N); transient plasmid expression in U2OS; as also suggested by Reviewer #3). In this way, we managed to confirm that the C-terminus faces the intermembrane space, while the N-terminus faces the matrix **(Fig. 3A, S3).** This aligns well with the results of the N-term BioID that detected TFAM and many other matrix proteins. The results of the C-term BioID could be explained by this topology as well, however, we now think that the C-terminal BirA tag hampers the interactions with nucleoids (see the reply to comment #8 of Reviewer #2), perhaps via interfering with protein folding, so we refrained from combining these two results in one conclusion and adjusted that section.

We also agree with the reviewer that our conclusions regarding sodium carbonate extractions were not properly formulated. According to the literature, a partial susceptibility to SC can be caused by the moderate hydrophobicity of the TM helix. We have additionally performed SC extraction at pH 9.5 and found c17orf80 to only pellet with the membranes under these conditions. We, therefore, conclude based on the facts that the C-terminal tag of c17orf80 fusion is detectable in the IMS, that the protein is resistant to SC at pH 9.5, and that it is partially but substantially resistant to SC at pH 11, that c17orf80 interacts with the IMM via its C-terminal TM helix. We have adjusted the respective section in the revised manuscript.

2. Basal enrichment of c17orf80 at nucleoid structures in untreated cells is not obvious in the images shown (fig. 2), and enlargements should be shown. Linescan analysis would also be helpful to demonstrate that c17orf80 indeed enriches at nucleoid structures, both in these images and after ddC/EtBr treatment (Fig. 4).

We thank the reviewer for this suggestion. We have changed the representation of IF images according to this recommendation and included linescans. In addition, we increased the intensity of the images where it was reasonable.

3. The complexome analysis is not convincing given the low abundance of c17orf80 detected (19 peptides) and the high background (10 peptides identified in the KO!). To confirm that c17orf80 is in a high molecular weight complex, the authors should perform blue native PAGE or related analyses.

We agree with the reviewer that the identification of c17orf80 peptides by our proteomic search was somewhat questionable, in particular for the peptides that showed up in the KO profiles. To make sure that these signals were just noise and not real identifications by MS/MS, we repeated the search but now with much more strict parameters and disabling the option for “matching between runs”. The latter allows the identification of extra peptides by using data from other LC-MS runs and more tolerance for a time window to assign unidentified features. This, of course, works much better for abundant proteins. When comparing the control profiles against the KO ones in a search without “matching”, we could see that only 7 peptides were identified in the control and no peptides for the KOs. Based on this, we are now sure that the signals (after removal of noisy peptides) are trustable and indicate that c17orf80 is indeed identified at high molecular mass. This information has now been added to the respective section.

Following the reviewer’s suggestion, we performed Western blotting of the native gel to identify c17orf80 and its location(s). In this case, we still used high-resolution clear native PAGE (hrCNE), instead of BN- PAGE, as it has recently been reported in another study from our group to improve the detection of protein complexes involving DNA-/RNA-interactions (https://www.biorxiv.org/content/10.1101/2023.04.03.534993v1), thus we prefer to use this gel type over Blue Native. Consistently with our complexome profiling analysis, we detected c17orf80 migrating at high molecular mass and, once again, validated the knockouts as no band of c17orf80 was seen. We have now included this blot as a supplementary figure **(Fig. S7A)**.

Since the complexome profiling analysis was based on hrCN-gels and the WB essentially showed the same pattern and matched the migration profile, we thus suggest that c17orf80 was correctly identified in that molecular mass range potentially interacting with other proteins present in the identified cluster.

4. The potential interaction between DNAJA3 and c17orf80 is not explored in any depth, nor are hit proteins from BioID analysis. Blue native page analysis could be used to test if DNAJA3 co-migrates

© 2023. Published by The Company of Biologists under the terms of the Creative Commons Attribution License (https://creativecommons.org/licenses/by/4.0/).

with c17orf80. Does loss of DNAJA3 affect c17orf80 migration size? The authors could also further confirm/explore a potential interaction between these and other proteins by immunoprecipitation and western.

The main outcome of our BioID experiment was the fact that c17orf80 interacts with nucleoid-associated proteins, such as TFAM, thus confirming its nucleoid localization detected by microscopy. Validation of other potential interactors was out of the scope of this paper since it would require functional analysis and we could not yet establish the function of c17orf80. In addition, we have our complexome dataset that offers unbiased identification of proteins, in our case thousands, in a single experiment. We believe that performing immunoprecipitation cannot be alone conclusive to prove or disprove a protein-protein interaction. Other methods, for instance, based on cross-linking, could offer a much better way to tackle these questions. However, they are time-consuming and beyond the scope at this point.

Regarding our findings on the potential interaction of c17orf80 and DNAJA3, our complexome profiling data suggests that the two proteins belong to a high molecular mass complex (around 3 MDa). Based on the size, it is reasonable to consider more components involved, but with the current resolution, we could not define whether those belong to the group of RNA-binding proteins, membrane-organization complexes or something else (we have now elaborated on this in the manuscript). In fact, we have also detected the comigration of DNAJA3 and c17orf80 in an independent complexome dataset generated with EtBr-treated cells, thus confirming the reproducibility of this finding **(Fig. S7D)** (as this dataset has recently been published as a preprint, we now mentioned this fact in the manuscript). We did not validate this interaction with an immunoprecipitation experiment, since both negative and positive results of co- IP alone would not be sufficient to prove the interaction between the two proteins or lack thereof. To answer this question, much more experimental work would be required, including functional analysis. Since both DNAJA3 and c17orf80 lack defined molecular functions, such experiments would deserve a separate manuscript. Our point was to show the potential interactors that might be associated with c17orf80, which may serve as a basis for future investigations. We have adjusted the respective parts of the results and discussion to make our intentions and conclusions more clear.

5. If the putative zinc finger motif is indeed matrix localized, is this motif required for enrichment at nucleoid structures? A more careful structure/function analysis of the protein could be a way to enhance the manuscript.

We agree with the reviewer that this information would be useful for understanding the nature of c17orf80. We nevertheless think that it would not substantially benefit this particular report. The scope of this paper is to introduce c17orf80 as a novel nucleoid-associated protein and to suggest possible hypotheses about its function for future research. As we still don’t know its exact molecular function and physiological role, we do not think it is reasonable to invest in this kind of experiment at this point. However, we will consider including a more detailed structural analysis with the follow-up studies.

Reviewer 2 Advance Summary and Potential Significance to Field...

This manuscript describes the cytological, functional and biochemical characterization of a putative mitochondrial nucleoid protein, c17orf80. The work is generally well done and this is a useful contribution to the field.

Reviewer 2 Comments for the Author...

I encourage revision of this work to consider the following; Essential to address:

1. Re evolutionary relationships of c17orf80, the text states that only potential homologs containing both zinc finger and ATP synthases homologous domains were considered. Are there other more distant homologs that contain one domain or the other?

Since only the terminal parts of the protein **(Fig. 1D)** are structured and conserved, we defined c17orf80 homologs by the simultaneous presence of the two domains. Individually, both domains

© 2023. Published by The Company of Biologists under the terms of the Creative Commons Attribution License (https://creativecommons.org/licenses/by/4.0/).

are found in many older eukaryotic species, however, they are not sufficient to define the origin of c17orf80. We have clarified this in the revised version.

2. Detection of the endogenous protein by immunofluorescence indicates that it is relatively abundant (comparable to e.g. TFAM). While it clearly colocalized with nucleoids and/or BrU foci under stressed conditions, the imaging as presented doesn't demonstrate a compelling homeostatic association.

We are not entirely sure what the reviewer means by a homeostatic association but we assume the reviewer meant that in the original IF images for the control, it was difficult to judge the C17orf80-mtDNA co-localization. For this reason (see also comment #2 by Reviewer #1 and our response) we have changed the representation of IF images to show this more clearly and present co-localization also by linescans as suggested by Reviewer #1 and by this reviewer (comment #4). In the revised Fig. 2 we likewise show the additional control antibody (against CypD) in much more detail clearly showing a more uniform IF signal compared to C17orf80 and mtDNA. The latter two show a very similar punctate pattern as also shown by the linescans. However, one of our points is that c17orf80 indeed better colocalizes with mtDNA under ddC/EtBr conditions, but shows a less pronounced punctate pattern in the control cells.

In case the reviewer meant to ask how abundant c17orf80 is compared to TFAM or other detected proteins, we have to note that our immunofluorescence microscopy approach is only semi-quantitative, as the IF signal intensity varies depending on the used primary antibody and fluorophores and their combinations, thus it is not possible to estimate a relative abundance of two proteins.

3. I would appreciate a rationale for the fixation conditions use in Figure 3. Could the authors provide some evidence that inner mitochondrial membrane structure is well-preserved?

The fixation conditions for Fig. 3 are identical to fixation conditions of all IF experiments using commonly used paraformaldehyde that has been used in numerous IF studies both for mitochondrial as well as any other type of human cell IF study. We assume the reviewer refers more to the use of the digitonin detergent that we use to lyse the outer membrane (and other membranes) selectively but not the inner membrane. This methodology is also reasonably well established, initially developed by J Lippincott- Schwartz (see doi: 10.1038/nmeth857), in combination with protease accessibility instead of antibody accessibility. Digitonin is a detergent that has selectivity for cholesterol-containing membranes hence explaining why the inner mitochondrial membrane, which has very little cholesterol, is relatively resistant to digitonin at the used concentration and incubation time. The intactness of the inner membrane is evident from the inaccessibility of the matrix proteins for antibody binding.

In the revised manuscript we now have used the digitonin antibody accessibility assay also for the determination of C17orf80 topology (see Reviewers' #1 and #3 comments) and have, along with these experiments, also included more matrix, OMM and IMS markers.

4. The images presented in Fig 4 are too zoomed out. I can't easily see colocalization or lack thereof. The authors should add linescans showing fluorescence intensity for each channel, further zoomed in cropped images, or both.

We found this suggestion very useful and have changed the representation of IF images according to this recommendation and increased the intensity of the images where it was reasonable.

5. In Fig 4 E there are horizontal lines across the cell nuclei which appear to be artifacts of either the microscopy or image processing. What is the explanation for this? Better quality images and raw data files need to be provided.

This is a microscope artefact that we see with rare images, typically only for one wavelength (in this case for DAPI). It only occurs in apotome mode and therefore it is likely caused by the mechanics (with moving grids) of the apotome system. Since in the revised version we now only show details of the full image for Figure 4 and the DAPI channel is not relevant or essential, this is

© 2023. Published by The Company of Biologists under the terms of the Creative Commons Attribution License (https://creativecommons.org/licenses/by/4.0/).

considered a moot point. On other rare occurrences during this study, it only affected the DAPI channel and therefore did not affect the analysis and interpretation of our results.

As requested, we are providing the microscopy raw and source files for both main and supplementary figures. As this is a lot of data, we uploaded the zipped folders with the organized files in SURFfilesender via our institutional access: https://filesender.surf.nl/?s=download&token=0b0cdf8b-e1d6-4ccd-8e84- e9e6754ec8a9 (link valid till 21/05/2023). This is a temporary storage and we would like to ask the editor whether sharing these data with only reviewers and the editor is sufficient, or they would recommend depositing these files for permanent access.

6. The methods re microscopy lack sufficient detail. What laser lines were used, what is the pixel size, etc. See https://pubmed.ncbi.nlm.nih.gov/20513764/ Fig 2 as a starting point.

The requested details are now included in the M&M section of the revised manuscript.

7. In the section 'C17orf80 accumulates in nucleoids upon short term treatment with ddC', data discussed including chloramphenicol, UV light and H2O2 treatment but are not shown. This is unacceptable. All data must be shown that are discussed.

The data are presented as a supplementary figure in the revised version **(Fig. S4)**.

8. Mitochondria appear fragmented upon overexpression of c17orf80-BirA-C term (Fig S5). Please discuss how this could contribute to the discrepancies in proteomic interaction networks between N term and C term tagged constructs presented in Fig 6.

We thank the reviewer for pointing this out. We more closely examined the localization of the fusion proteins and realized that although the C-terminal BirA fusion was imported to mitochondria, its expression was rather abnormal. After transient transfection with this construct, we found many cells with a highly fragmented mitochondrial network where c17orf80-BirA-C was not colocalized with mtDNA. Also, in cells with a normal mitochondrial network, c17orf80-BirA-C was often distributed uniformly without enriching at nucleoids (this was not the case for BirA-N- and Myc-C-tagged fusions). Although we also found rare cells where C17orf80-BirA(C) showed a proper punctate pattern (see the Figure below) and it is, therefore, possible that in the stably-expressing FlpIn cells, c17orf80-BirA-C protein still interacts with the nucleoids, we have no way to confirm this. Therefore, we decided to focus more on the results of the N-terminal BioID **(Fig. 6)** and to note that the results of the C-terminal BioID can be explained by the interference of the tag with the protein localization. We also added the comparison of the expression patterns and mitochondrial network morphology in cells transiently expressing the c17orf80-BirA fusions **(Fig. S6)**.

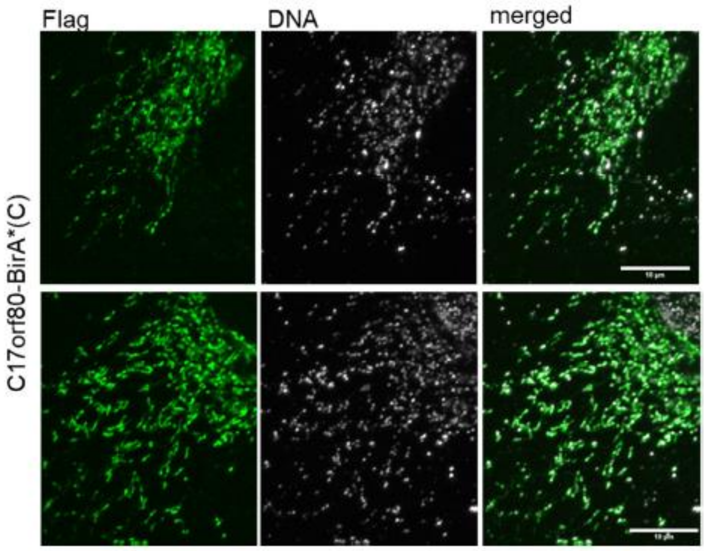

© 2023. Published by The Company of Biologists under the terms of the Creative Commons Attribution License (https://creativecommons.org/licenses/by/4.0/).

U2OS cells transfected with C17orf80-BirA(C) construct and detected with antibodies against Flag-tag and DNA. The upper panel shows a cell with the normal mitochondrial morphology where c17orf80- BirA(C) colocalizes with mtDNA. The lower panel shows a cell with fragmented mitochondrial network and more uniform c17orf80 signal.

9. The proper control for the proteomics presented in Fig 6 would be enrichment of nucleoid interactions as compared to a soluble matrix protein like eGFP. Was this normalization done? Perhaps I missed this.

We do not entirely agree with this statement. A soluble BirA-tagged matrix protein can perhaps be considered the most stringent control, but it is questionable if it is 'the' proper control. One might argue that a soluble matrix protein that is overexpressed would be able to biotinylate many mitochondrial proteins inappropriately, including many factors involved in mitochondrial gene expression. It would therefore increase stringency but decrease sensitivity, which is always one of the considerations to make in deciding which controls to use. Notwithstanding this discussion, we here controlled for non- specific biotinylation using non-induced cells cultured with the addition of biotin. Normalization thus was done with four biological repeats comparing uninduced and induced C17orf80-BirA fusions, all in presence of added biotin, which yielded a plausible list of potential interactions. Taking into account the fact that BioID pulldowns with C-term and N-term fusions provided distinct results, we are convinced that our experimental setup and the results are accurate. For the readers' convenience, the control is now also clearly specified in the legend of Fig 6.

10. The description of c17orf80 as a "nucleoid resident" protein in the Discussion is overstated, given the data.

We have replaced this term with "nucleoid-associated protein".

Preferred, but not essential to address:

c17orf80 has two TM domains; I'm curious whether both are required for enrichment of the protein with nucleoid structures. Perhaps the authors could transiently express truncation mutant(s) from plasmids in the KO cells.

This is indeed an appropriate question to ask, as is the question of whether or not the putative zinc finger is involved, as commented on by Reviewer #1. We, however, find these experiments beyond the scope of our current work, which establishes c17orf80 as a novel nucleoid-associated protein. We will hopefully be able to address these questions in a follow-up study.

Reviewer 3 Advance Summary and Potential Significance to Field...

In their work, Potter et al. define the localization of c17orf80 and endeavor to characterize its function. This protein has previously been classified as resident in mitochondrial RNA granules by high- throughput approaches coupling proximity labelling and mass-spectrometry. Using the same approach, the authors convincingly show that due to the predicted topology of c17orf80, N-terminal tagging of the protein (compared here with the previously employed C-terminal tagging) yields a more accurate assignment of c17orf80 as a peripheral component of mitochondrial nucleoids. This assignment is further supported by biochemical fractionation and mitochondrial complex profiling. Immunofluorescence imaging using a commercial c17orf80 antibody shows the co-localization of the signal with mitochondria, TFAM and mitochondrial DNA. Interestingly, the authors observe that c17orf80 puncta increase in intensity when mtDNA replication is stalled by treatment with ddC, a cytosine analogue. Despite extensive investigation of the state of mtDNA following c17orf80 knock-out and knock-down, the authors haven't been able to assign a function for c17orf80 in cultured U2OS cells.

Reviewer 3 Comments for the Author...
Generally, I find this study well performed and data accurately interpreted. Given that it definitively addresses apparent inconsistencies in the high-throughput assignment of c17orf80, I recommend it for publication in JCS.

© 2023. Published by The Company of Biologists under the terms of the Creative Commons Attribution License (https://creativecommons.org/licenses/by/4.0/).

Major comments:

1. All immunofluorescence-based results in this study rely on the commercial c17orf80 antibody.

Although the authors were able to validate the antibody using knock-down (Fig S2), they remark on variability in intensity and clear punctate background staining which persists after c17orf80 knock-down (Fig S2, p7). To strengthen key conclusions about c17orf80 localization, it is necessary to confirm the immunofluorescence data using an orthogonal imaging strategy. The authors could consider overexpressing a fluorescently tagged c17orf80, or perform immunofluorescence on the BirA-flag-fusion expressing cells.

We agree that this is an important validation step. Accordingly, we have used a transiently expressed Myc-tagged c17orf80 detected with an anti-Myc antibody to confirm the IF pattern **(Figure S2B)**. In addition, we have detected the BirA-Flag fusions to validate their submitochondrial localization with an anti-Flag antibody **(Fig. S6B)**. Here, c17orf80-BirA-N fusion was also clearly co-localized with mtDNA.

2. The conclusion of this study could be strengthened by performing the following experiments, although these are not strictly required:

a. In addressing comment 1, the authors might consider performing the antibody accessibility test akin to Fig 3A on stained BirA-flag-fusion expressing cells. This could be a tractable way to experimentally test c17orf80s topology.

We thank the reviewer for this suggestion. We performed the antibody accessibility assay with all three constructs we had (x2 C-terminal tags and x1 N-terminal tag) and found that the C-terminal tags are exposed to the intermembrane space, while the N-terminal tag cannot be detected without complete permeabilization of the membranes, confirming that the N-terminus of the protein faces the matrix, and the C-term TM helix anchors the IMM. We added this information to the respective section and **Figures 3 and S3.** We also validated that the signal from the C-terminal tags we observed was indeed mitochondria-derived and that IMS proteins were detectable under our partial lysis conditions.

b. The authors remark on variability of c17orf80s behavior following carbonate extraction (p7), and later they observe c17orf80 relocalization following ddC treatment. It might be worthwhile to repeat the carbonate extraction following ddC treatment to assess whether replicative status might have contributed to the experimental variability shown in Fig 3.

We have performed two experiments in this direction: SC extraction with ddC-treated cells and c17orf80-BioID-N with ddC-treated cells. Surprisingly, we did not detect any substantial change in c17orf80 distribution or interactors. We decided not to include this data in the manuscript as it does not contribute to its conclusions. We provided the data for both experiments for the reviewer below. Perhaps, a stronger treatment would provide more information, we are considering repeating these experiments with EtBr.

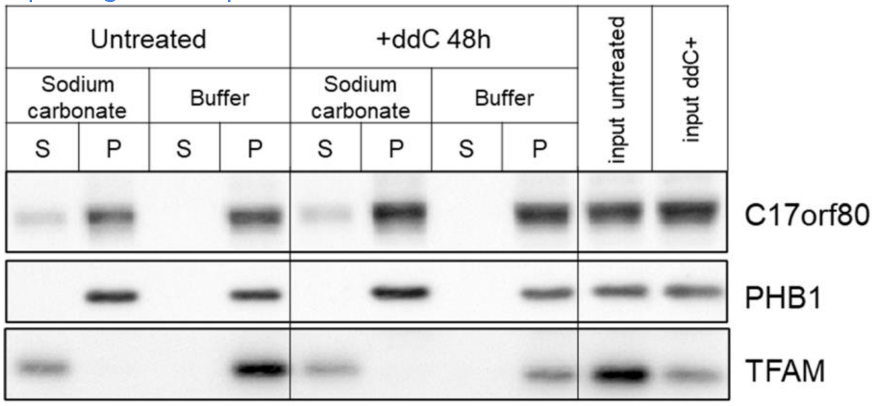

© 2023. Published by The Company of Biologists under the terms of the Creative Commons Attribution License (https://creativecommons.org/licenses/by/4.0/).

Sodium carbonate extraction at pH 11 using mitochondria isolated from control and ddC-treated cells (HEK293, 100 uM ddC for 48h). The distribution of c17orf80 between pellet and supernatant fractions was no substantially affected. A moderate increase in c17orf80 amount was observed in ddC-treated sample.

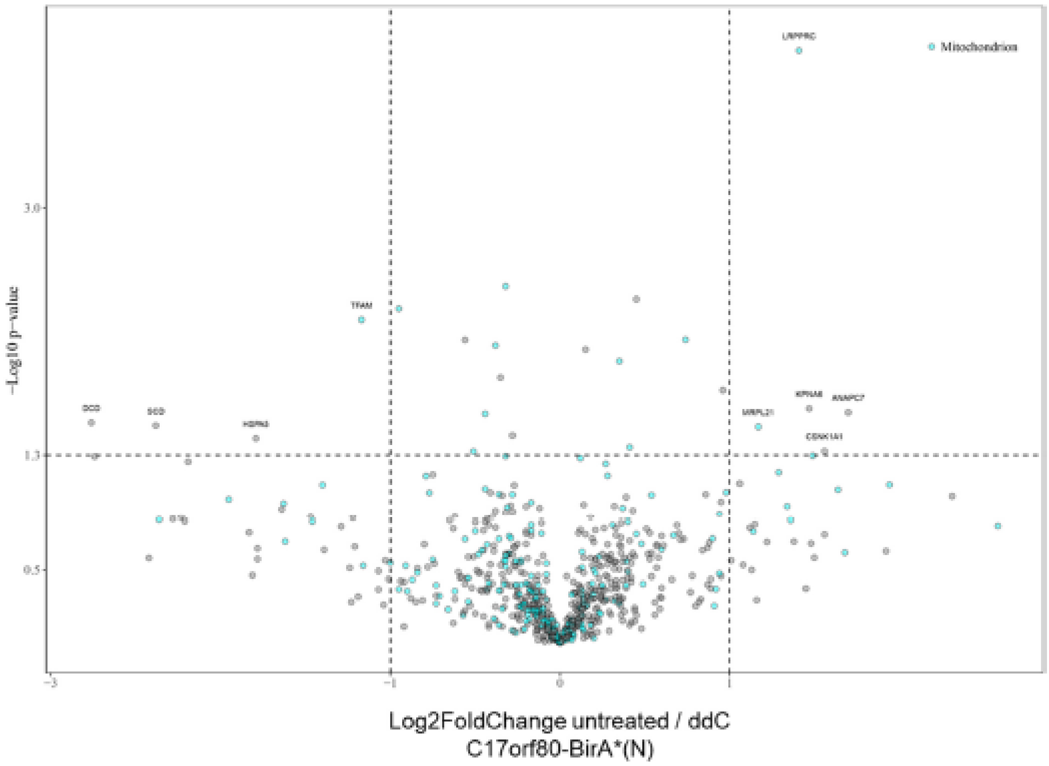

Comparison of BioID interactomes obtained with C17orf80-BirA(N)-expressing cells treated or not with ddC for 48h. The experimental and visualization details are the same as on Fig. 6A. The protein identifications were overall very similar. The ddC pulldown showed less enrichment for TFAM (2.25- fold), which reflects the reduction of mtDNA in response to ddC-treatment, and higher enrichment for LRPPRC (2.65-fold). We checked whether LRPPRC co-localizes with c17orf80 in control or ddC-treated cells and found that they do not, so the increase in LRPPRC can also be a response to ddC-treatment and not a reflection of an increased interaction with c17orf80.

c. The conclusion about c17orf80 interaction with DNAJA3 drawn from the mitochondrial complex profiling would be strengthened by direct IP. Although DNAJA3 but not c17orf80 was found to co- immunoprecipitate with nucleoids in the past, it is possible that their interaction could be detected biochemically.

We totally agree with the Reviewer that more proof would be required to demonstrate a functional and possible direct physical association between C17orf80 and DNAJA3. But co-IP, although relatively quick to perform, we don't consider as a very reliable method. Apart from the fact that it would often again require overexpression of tagged variants of proteins, proteins that have a strong association with nucleic acid are highly prone to give co-IP artefacts by an indirect association of proteins in protein nucleic-acid complexes. In contrast, the complexome profiling (CP) method we have applied only examines endogenous proteins/protein complexes and in our case was used with prior treatment of mitochondrial lysates with DNAse I to degrade mtDNA. Resulting co-migration patterns suggest proteins to be present in the same complex, but by no means prove it. Similar responses to various treatments do provide a strong indication though, and for this reason, we have now also included CP data extracted from a recently published complexome generated with EtBr-treated cells. There, DNAJA3 and c17orf80 are clearly comigrated **(Fig. S7D)** (as this dataset has recently been published as a preprint, we now mentioned this fact in the manuscript).

© 2023. Published by The Company of Biologists under the terms of the Creative Commons Attribution License (https://creativecommons.org/licenses/by/4.0/).

Nevertheless, even if two very distinct methods such as CP and BioID have identified DNAJA3 as a potential partner for C17orf80, we agree that the evidence is circumstantial, and now adjusted the section to put less emphasis on it. We feel it is an interesting entry point for future research on the function of C17orf80 but beyond the scope of the current manuscript to address it experimentally.

Minor comments:

1. The authors should include data for intensity/relocalization of c17orf80 upon other mitochondrial stressors (rather than 'data not shown' on p9).

We have included these data in supplemental figures **(Fig. S4)**.

2. The authors quote the H(ES) value for c17orf80 but not DNAJA3 abundance variation in complex profiling performed in control vs ddC treated cells. This would be a helpful to support the conclusion that the two proteins interact.

We have included this information in the respective part of the text. In addition, in the revised version, we have rephrased the part about the interaction between DNAJA3 and c17orf80 to avoid any confusion. Our intention was to indicate their similar behaviour on the native gel accompanied by the BioID data, but not to state these two are true interactors.

3. The labels on top of Volcano plots in Fig 6A-B are confusing. Given the authors postulate there is a biologically significant interaction between c17orf80 and DNAJA3, it would be pertinent to label DNAJA3 in Figure Fig 6A-B in addition to clarifying the labels.

We have simplified the respective figure **(Fig. 6)** by removing the volcano plots showing the results of the C-terminal BioID from the main figure as we reconsidered the validity of these results. These data can now be found in the Supplemental Data 2 file. We have adjusted the labels and titles of the c17orf80-BirA-N volcano plot according to the reviewer's recommendation.

4. P13, line 20 refers to KO validation by western blot without a figure reference. Did the authors mean to refer to Fig 8A?

We have fixed this and referred to **Figure 8A** and also to the newly added Western blot of the native gel on **Figure S7A.**

5. It would be helpful to include a color-code for Fig 7A in the figure as opposed to figure legend only. Additionally, c17orf80 should be differentiated from the pink protein-modifying enzymes.

We have adjusted **Figure 7A** accordingly.

6. Figure legends for Fig S8A and S8B are swapped.

This has been fixed, thank you for noticing.

<u>Second decision letter</u>

MS ID#: JOCES/2022/260822

MS TITLE: Uncharacterized protein c17orf80: A novel interactor of human mitochondrial nucleoids

AUTHORS: Alisa Potter, Anu Hangas, Steffi Goffart, Martijn A. Huynen, Alfredo Cabrera-Orefice, and Johannes N. Spelbrink
ARTICLE TYPE: Research Article

© 2023. Published by The Company of Biologists under the terms of the Creative Commons Attribution License (https://creativecommons.org/licenses/by/4.0/).

I am happy to tell you that your manuscript has been accepted for publication in Journal of Cell Science, pending standard ethics checks. Please make the small correction to the text raised by reviewer 1.

Reviewer 1

Advance summary and potential significance to field

The authors have satisfactorily addressed all my concerns, and importantly have now conclusively demonstrated the topology of c17orf80. I recommend the manuscript for publication.

Comments for the author

I did notice a few typos on lines 132-138, where figure references are made to Figure S4 rather than Figure S3 that should be corrected.

Reviewer 2

Advance summary and potential significance to field

This manuscript describes the cytological, functional and biochemical characterization of a putative mitochondrial nucleoid protein, c17orf80. The work is generally well done and this is a useful contribution to the field.

Comments for the author

The revised manuscript is suitable for publication. I have no further comments.

Reviewer 3

Advance summary and potential significance to field

The authors have addressed my major comment and performed additional experiments to strengthen the conclusions of their work. Although they were not able to show a direct interaction between c17orf80 and DNAJA3, they added new complexome profiling data and softened the language in the main body of the text to address my and the other reviewerÂ’s concern. Therefore, I am happy to recommend the manuscript for publication in its current form.

Comments for the author

See above.

© 2023. Published by The Company of Biologists under the terms of the Creative Commons Attribution License (https://creativecommons.org/licenses/by/4.0/).
